# Spatial epidemiology of eastern equine encephalitis in Florida

**DOI:** 10.1186/1476-072X-11-47

**Published:** 2012-11-05

**Authors:** Patrick T Vander Kelen, Joni A Downs, Lillian M Stark, Rebecca W Loraamm, James H Anderson, Thomas R Unnasch

**Affiliations:** 1 Global Health Infectious Disease Research Program, University of South Florida, 3720 Spectrum Blvd, Tampa, FL, 33612, USA; 2 Department of Geography, Environment, and Planning, University of South Florida, 4202 E. Fowler Ave, Tampa, FL, 33620, USA; 3 Florida Department of Health, Bureau of Laboratories-Tampa, 3602 Spectrum Blvd, Tampa, FL, 33612, USA

**Keywords:** Eastern equine encephalitis, GIS, Spatial epidemiology, Compositional analysis, Euclidean distance

## Abstract

**Background:**

Eastern Equine Encephalitis virus (EEEV) is an alphavirus with high pathogenicity in both humans and horses. Florida continues to have the highest occurrence of human cases in the USA, with four fatalities recorded in 2010. Unlike other states, Florida supports year-round EEEV transmission. This research uses GIS to examine spatial patterns of documented horse cases during 2005–2010 in order to understand the relationships between habitat and transmission intensity of EEEV in Florida.

**Methods:**

Cumulative incidence rates of EEE in horses were calculated for each county. Two cluster analyses were performed using density-based spatial clustering of applications with noise (DBSCAN). The first analysis was based on regional clustering while the second focused on local clustering. Ecological associations of EEEV were examined using compositional analysis and Euclidean distance analysis to determine if the proportion or proximity of certain habitats played a role in transmission.

**Results:**

The DBSCAN algorithm identified five distinct regional spatial clusters that contained 360 of the 438 horse cases. The local clustering resulted in 18 separate clusters containing 105 of the 438 cases. Both the compositional analysis and Euclidean distance analysis indicated that the top five habitats positively associated with horse cases were rural residential areas, crop and pastureland, upland hardwood forests, vegetated non-forested wetlands, and tree plantations.

**Conclusions:**

This study demonstrates that in Florida tree plantations are a focus for epizootic transmission of EEEV. It appears both the abundance and proximity of tree plantations are factors associated with increased risk of EEE in horses and therefore humans. This association helps to explain why there is are spatially distinct differences in the amount of EEE horse cases across Florida.

## Background

Eastern Equine Encephalitis virus (EEEV) is a highly pathogenic arbovirus endemic to North, Central, and South America. The mortality rate for symptomatic cases of EEE is 35% or more with survivors facing disability from neurological sequelae [1]. From 1964–2010, human cases of EEEV were reported in 20 U.S. states [2], with Florida being the most affected, accounting for 25% of all reported human fatalities to EEE. The enzootic transmission of EEEV is maintained in a mosquito-avian cycle predominantly involving the vector *Culiseta* (*Climacura*) *melanura* (Coquillett) and passerine birds [3,4]. The epizootic cycle of EEEV involving humans and horses involves bridge vectors that are known to feed on both avian and mammalian hosts. Documented and proposed bridge vector species include *Aedes* (*Aedimorphus*) *vexans* (Meigen), *Coquillettidia* (*Coquillettidia*) *perturbans* (Walker), *Culex* (*Melanoconion*) *erraticus* (Dyar and Knab), *Culex* (*Culex*) *nigripalpus* Theobald, *Ochlerotatus* (*Ochlerotatus*) *canadensis* (Theobald), and *Ochlerotatus* (*Ochlerotatus*) *sollicitans* (Coquillett) [5-7]. Enzootic EEEV transmission has been associated with hardwood swamp habitats [8] and tree plantations [9]; however, little is known about the ecological associations in the epizootic transmission sites.

EEE is a reportable human and veterinary disease in the United States [10]. In the northeast and south central states, epizootic outbreaks involving humans and horses peak in August and September [11]. In contrast, EEEV transmission in Florida occurs throughout the year, with most human and horse cases occurring in June and July [12]. From 2005–2010, the United States had 1380 horse fatalities from EEE, of which 442 were in Florida (32%) [13]. Despite the availability of an effective equine EEEV vaccine, Florida averages 70 EEEV equine case fatalities per year. Currently there is no approved vaccine for humans or effective medical treatment for those infected with the virus. Prevention strategies to protect the human population from EEE thus rely primarily upon case detection and vector control.

In previous studies, spatial methods were used to associate particular habitats with seroconversions of sentinel chickens to EEEV in Walton County, Florida [9]. Because EEEV is maintained in an enzootic cycle involving passerine birds as the vertebrate reservoir and chicken sentinels attract ornithophilic mosquito species that serve as the enzootic vectors for the virus, this study primarily assessed habitats associated with the enzootic cycle. Through the use of spatial epidemiology, this research aims to improve our understanding of the ecology of EEEV in Florida by examining the spatial distribution and habitat associations of documented horse EEE fatalities through examining habitats associated with the epizootic cycle in which mammals are exposed to the virus.

Spatial epidemiology is the study of the geographical variation in disease risk or incidence [14]. As a growing field, spatial epidemiology provides new insights into arbovirus transmission as it pertains to environmental interactions. Geographic Information Systems (GIS) and remote sensing are just a few of the tools used to measure spatial variation in disease risk [15-19]. In terms of arthropod-borne diseases, GIS has been employed to analyze environmental factors associated with Lyme borreliosis [20,21], tick-borne encephalitis [22], West Nile virus [23-26], Dengue virus [27,28], and Eastern Equine Encephalitis virus [9,29]. Spatial clustering is a GIS technique routinely utilized to explore patterns of disease transmission. Identifying the geographical location and distribution of disease allows researchers the opportunity to analyze the potential local or regional drivers of disease transmission. Research has shown that areas with spatial clustering of vectors and hosts may increase the risk of disease transmission [30]. Spatial clustering methods have also successfully been used to detect high risk areas for West Nile virus [15,31] and Ross River virus [32]. This study applies clustering and other spatial epidemiological techniques using GIS to understand the spatial variation in horse cases of EEEV in Florida. The main goals of the research were to: (1) identify counties with the highest incidence rates of EEE in horses, (2) explore regional and local clusters of EEE horse fatalities, and (3) determine habitats associated with EEEV in horses, in terms of both abundance and spatial proximity.

## Results

### Incidence

**Figure 1:**
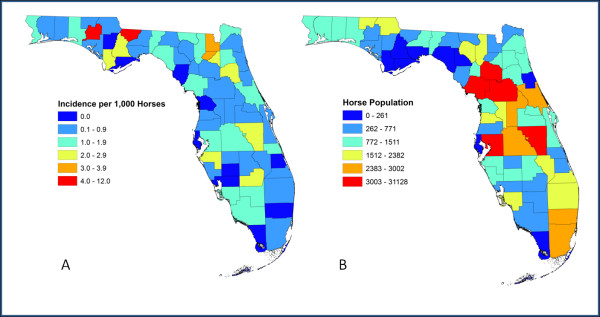
**Cumulative incidence rates per county per year.A**) County based cumulative incidence from 2005–2010 per 1,000 horses per year normalized by population. **B**) County based horse populations from 2007 Census.

Florida contained a total of 120,614 horses according to the 2007 equine census data [33], with all but two counties having horses. The highest density of horses occurred in the Northern region of Florida. County based cumulative incidence rates of EEE for 2005–2010 varied across the state. The average incidence rate per county per year was 1 case of EEE per 1,000 horses. Fourteen counties had cumulative incidence rates of 2 cases per 1,000 horses per year or higher with 10 of the 14 being in the Northern region (Figure 1). Washington County, located in the Panhandle region, had the highest incidence rate of EEE cases at 12 per 1,000 horses per year. The area with the lowest incidence rates was the Southern region, despite the fact that 4 of the 7 counties reported horse cases. Ten counties had no horse cases during 2005–2010, of which 8 were coastal counties. The results of the Spearman’s rank correlation coefficient showed that there was no significant relationship between the number of cases and the total population of horses in each county (ρ = 0.24, p = 0.06). Washington County had the highest incidence of disease while having one of the lowest county horse populations, while Marion County had a large population of horses and a low incidence rate.

### Cluster analysis

**Figure 2:**
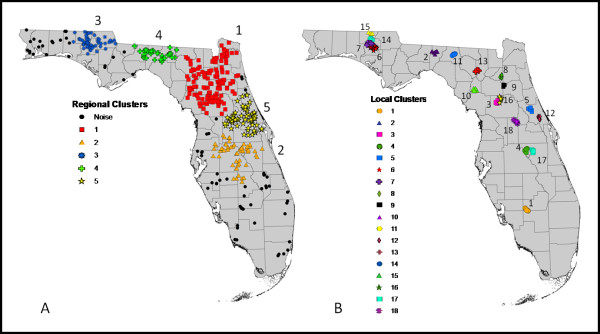
**Density-Based Spatial Clustering of Applications with Noise for EEE cases in Florida from 2005–2010.A**) Regional clustering with 5 clusters. **B**) Local clustering with 18 clusters.

DBSCAN identified five regional EEE case clusters across Florida during 2005–2010 (Figure 2). Case clusters included 360 out of the 438 cases with the remaining 78 cases being identified as statistical noise. The largest clusters were Cluster 1 in North Florida (145 cases) and Cluster 5 (66 cases) in the Central Region (Table 1). Cluster 1 had contributing cases every year, averaging 24 cases per year with a maximum of 51 in 2005 and a minimum of 4 in 2007. Cluster 5 had an average of 11 cases per year with a maximum of 25 in 2005 and a minimum of 2 in 2006 and 2007. The smallest Cluster was cluster 4 which had 33 cases over all, with no contributing cases in 2007. The most productive year for EEE cases in the regional clusters was 2005, with 120 cases included.

**Table 1:** Regional clusters with cases by year and cluster

**Cluster**	**2005**	**2006**	**2007**	**2008**	**2009**	**2010**	**Total**
1	51	8	4	29	40	13	145
2	31	1	4	5	1	16	58
3	8	0	6	26	5	13	58
4	5	3	0	9	9	7	33
5	25	2	2	14	12	11	66
Total	120	14	16	83	67	60	360

DBSCAN identified 18 local clusters in 17 different counties throughout Florida. A total of 105 (24%) of all cases were within the local clusters (Table 2). Ten of the 18 clusters were located in the North region of Florida, with only one cluster found in the South. Five of the seventeen counties (Holmes, Washington, Marion, Volusia, and Osceola) had two local clusters within their boundaries. None of the local clusters had consistent yearly case contributions for all six years. However, the average cluster had cases in three of the six years. Cluster 7 had 11 contributing cases out of the 105 and had activity in four of the six years. Clusters 6 and 7 had a combined sum of 20 cases representing 19% of the total cases within the local clusters; both of these clusters were located in Washington County. The most productive years for EEE cases in the local clusters were 2005 and 2008, both years reporting 30 cases (Table 2).

**Table 2:** Local clusters with cases by year and cluster

**Cluster**	**2005**	**2006**	**2007**	**2008**	**2009**	**2010**	**Total**
1	2	1	0	0	0	3	6
2	2	0	0	2	0	2	6
3	2	0	1	0	0	1	4
4	2	0	0	1	0	2	5
5	0	0	0	2	5	1	8
6	0	0	1	3	0	5	9
7	2	0	0	6	1	2	11
8	1	0	0	3	2	0	6
9	2	0	0	1	1	0	4
10	4	0	0	0	1	0	5
11	1	0	0	0	3	0	4
12	2	0	0	3	2	0	7
13	0	0	0	2	3	0	5
14	0	0	0	2	1	1	4
15	1	0	1	2	0	0	4
16	5	1	0	2	0	0	8
17	2	0	0	1	0	1	4
18	2	2	0	0	0	1	5
Total	30	4	3	30	19	19	105

### Habitat analysis

The predominant habitat in terms of abundance around the cases was cropland pastureland, comprising 25% of the area within the buffers. Tree plantations were the second most abundant feature (at 15%) with low density residential land shortly behind at 12%. Wetland coniferous forest, wetland hardwood forest, vegetated non-forested wetland and wetland forested mixed collectively comprised 18% of the habitat within the buffers (Table 3). The habitat compositional analysis revealed that five land cover classes were proportionally more abundant in the buffer area of the horse cases than in the surrounding landscape. The top five classes, in rank order, included: (1) low density residential, (2) crop and pastureland, (3) upland hardwood forest, (4) vegetated non-forested wetlands, and (5) tree plantations (Table 4). Six categories—urban, water, medium density residential, wetland hardwood forest, shrub and brushland, and mining—were less abundant in the buffers than in the surrounding landscape.

**Table 3:** **Proportions of habitat types within the 1**.**5km buffer area of EEE horse cases in Florida**

**Habitat**	**Area (ha)**	**Percentage**
Crop and Pastureland	75895	25
Tree Plantations	45990	15
Low Density Residential	36436	12
Upland Hardwood Forest	28343	9
Medium Density Residential	19688	6
Upland Coniferous Forest	17314	6
Urban	12783	4
Wetland Forested Mixed	15298	5
Vegetated Non-Forested Wetland	14069	5
Wetland Hardwood Forest	13860	4
Wetland Coniferous Forest	11950	4
Water	9589	3
Shrub and Brush land	5315	2
Mining	2308	1
Total	308838	100

**Table 4:** Habitat compositional analysis

	**LR**	**CP**	**UHF**	**VNFW**	**TP**	**WFM**	**UCF**	**WCF**	**U**	**W**	**MR**	**WHF**	**SB**	**M**	**Rank**
LR	0	+++	+++	+++	+++	+++	+++	+++	+++	+++	+++	+++	+++	+++	1
CP	- - -	0	+++	+++	+++	+++	+++	+++	+++	+++	+++	+++	+++	+++	2
UHF	- - -	- - -	0	+++	+++	+++	+++	+++	+++	+++	+++	+++	+++	+++	3
VNFW	- - -	- - -	- - -	0	+	+++	+++	+++	+++	+++	+++	+++	+++	+++	4
TP	- - -	- - -	- - -	-	0	+	+	+	+++	+++	+++	+++	+++	+++	5
WFM	- - -	- - -	- - -	- - -	-	0	+	+	+++	+++	+++	+++	+++	+++	6
UCF	- - -	- - -	- - -	- - -	-	-	0	+	+	+++	+++	+++	+++	+++	7
WCF	- - -	- - -	- - -	- - -	-	-	-	0	+	+	+	+++	+++	+++	8
U	- - -	- - -	- - -	- - -	- - -	- - -	-	-	0	+	+	+	+	+++	9
W	- - -	- - -	- - -	- - -	- - -	- - -	- - -	-	-	0	+	+	+	+++	10
MR	- - -	- - -	- - -	- - -	- - -	- - -	- - -	- - -	-	-	0	+	+	+	11
WHF	- - -	- - -	- - -	- - -	- - -	- - -	- - -	- - -	-	-	-	0	+	+	12
SB	- - -	- - -	- - -	- - -	- - -	- - -	- - -	- - -	-	-	-	-	0	+	13
M	- - -	- - -	- - -	- - -	- - -	- - -	- - -	- - -	- - -	- - -	-	-	-	0	14

Simplified ranking matrices with 14 land classifications ranked in order of proportional habitat use between horse cases and the surrounding county (+ preference, - avoidance, a triple sign represents significant deviation from random at P < 0.05). LR=low density residential, CP=crop and pastureland, UHF=upland hardwood forest, VNFW=vegetated non-forested wetland, TP=tree plantations, WFM=wetland forested mixed, UCF=upland coniferous forest, WCF=wetland coniferous forest, U=urban, W=water, MR=medium density residential, WHF=wetland hardwood forest, and SB=shrub and brush land, and M=mining.

### Distance analysis

Euclidean distance analysis was applied in order to measure the spatial proximity of different habitats to EEE horse cases. Since the resulting distances were not normally distributed, they were summarized by medians rather than means (Table 5). EEE horse cases were on average closest to low density residential land (30-79m) and crop and pastureland (90-120m). Other habitats showed slight differences in proximity rankings, with upland hardwood forest, vegetated nonforested wetland, and tree plantations tending to be the next nearest habitats. Cases were on average 152–1034 m from upland hardwood forest, significantly closer than expected for all four regions. The median distances for vegetated nonforested wetland were 346–681 m, although these distances were either insignificant or significantly farther than expected based on the configuration of the surrounding landscapes. Cases averaged 248–694 m from tree plantations, significantly closer than expected in all but the North region. Horse cases were located farther from to the other habitat types, with various wetland types tending to be the next proximal, although the distances vary widely by region.

**Table 5:** Euclidean distances from habitats to cases based on ecological regions

**Habitat**	**Central**			**North**			**Panhandle**			**South**		
	**Horse**	**Region**	***p***	**Horse**	**Region**	***p***	**Horse**	**Region**	***p***	**Horse**	**Region**	***p***
LR	78.5	1879	<0.001	60	494	<0.001	75.5	254	0.001	30	8470	0.0002
CP	114	150	0.65	94	416	<0.001	90	390	<0.001	120	1373	0.002
UHF	516.5	834	0.003	182	488	<0.001	152	432	<0.001	1034	2698	0.001
VNFW	346.5	240	<0.001	408	421	0.005	426.5	421	0.390	618	201	0.001
TP	693.5	1106	<0.001	258	127	<0.001	248	108	<0.001	680	6577	0.001
WFM	868	1874	<0.001	569	494	<0.001	211	254	0.085	3977	8470	0.753
UCF	744.5	1120	0.046	524	831	<0.001	439	576	0.141	1290	6332	0.075
WCF	709.5	1574	0.001	715	757	0.019	390	523	0.233	576	2782	0.507
U	705.5	1449	<0.001	751	1317	<0.001	672	1341	<0.001	964	3360	0.001
W	362.5	550	0.002	630	982	<0.001	553	807	<0.001	540	1281	0.023
MR	1383	3156	0.001	2280	3161	<0.001	660	2735	<0.001	2093	8912	0.023
WHF	664	597	0.002	600	593	0.001	653	882	0.334	865	543	0.013
SB	883	1149	0.778	1221	1465	0.142	1095	1410	0.121	1425	3502	0.55
M	2177	2694	0.326	2057	2731	0.005	1714	2340	0.052	1712	3750	0.152

Median Euclidean distances in meters for the four regions in Florida. Results from the Wilcoxon test with the p-value being significant at the 0.0036 after the Bonferroni correction. Horse = median (meters) from horse cases to habitat type. Region = median (meters) from random possible points to habitat type. LR=low density residential, CP=crop and pastureland, UHF=upland hardwood forest, VNW=vegetated non-forested wetland, TP=tree plantations, WFM=wetland forested mixed, UCF=upland coniferous forest, WCF=wetland coniferous forest, U=urban, W=water, MR=medium density residential, WHF=wetland hardwood forest, and SB=shrub and brush land, and M=mining.

## Discussion

Regional clustering of EEE horse cases highlighted the spatial differences of EEE transmission in Florida. The regional case clusters included 360 out of the 438 cases, which illustrates a spatial component in the transmission of EEEV to horses. The northern region accounted for 46% of the total horse cases from 2005–2010, as well as exhibited the highest incidence rates. In low transmission years, case clustering mainly takes place in the northern region of Florida. The regional clustering focuses on the inland counties reinforcing the lack of cases in the coastal counties, which are more dominated by saltwater marshes where EEEV is not endemic.

The local clustering highlights the focality of EEE transmission to horses and the density of cases within specific counties. The spatial location of the local clusters varied across the state. The most densely clustered area was in the panhandle region in Washington and Holmes counties. This area had four localized clusters, accounting for 29 out of the possible 106 cases and had cluster-contributing cases in all years except 2006. Local clusters were present in 18 of the 67 counties with 4 counties having more than one cluster. The northern region contained 10 of the 18 local clusters, which implies a strong focal nidus of transmission in this region. Results from the DBSCAN clustering method supports previous findings in which EEEV amplification was related to localized ecological conditions [34].

The finding that cropland and pastureland were the most abundant habitats surrounding equine cases of EEE was not surprising since that is where horses are typically found. The same can be said for low density residential areas, since it is the rural communities that have enough land area to support horse populations. Tree plantations, comprising 15% of the area around EEE cases, were found to be the next most abundant habitat associated with equine cases. The median distance of tree plantations from horse cases revealed that 50% of the cases fell within 470 m—significantly closer than expected for all regions in the state—suggesting that the proximity of tree plantations surrounding EEE cases may be an important factor in EEEV transmission to horses. The compositional analysis confirmed that tree plantations were over-represented in the EEE case buffers compared to its availability in the surrounding area.

Tree plantations seem to be an important ecological factor in EEEV transmission to horses in Florida. Previous studies in Walton County Florida have shown that tree plantations were associated with enzootic EEEV transmission [9]. The enzootic cycle of EEEV transmission involves avian hosts and the vector *Cs*. *melanura*; while the epizootic cycle involves equines and humans and various possible bridge vectors. The association of tree plantations with the risk of EEEV transmission in both sentinel chickens and horses suggests that tree plantations might harbor enzootic foci from which EEEV emerges into its epizootic cycle. One explanation is that the tree plantations often have a higher number of trees per hectare compared to other forest types [35]. The density and availability of these trees may make the habitat more attractive to nesting and roosting birds, and thereby increasing the intensity of both enzootic and epizootic EEEV activity. The tree density may also provide suitable sheltering locations for various mosquito species. The location of crop and pasturelands next to the tree plantations also might provide an edge effect, allowing for a greater concentration of both vector and avian populations within the horse habitats [36]. A concurrent explanation is that the tree plantation habitats often rest on poorly drained soil. The poor soil drainage could result in the inundation of the area, thereby creating temporary wetland conditions in close proximity to horses. Previous studies have shown that hydrologic conditions due to variations in temperature and rainfall can influence arbovirus vectors and hosts resulting in increased risk for dispersal into the surrounding areas [37,38].

Upland hardwood forests were found to be both significantly closer to horse cases then random (p<0.003) in all four regions. This suggests that the having upland hardwood forests located near areas with horses might be associated with a greater risk for EEEV transmission. The close proximity of cases to upland hardwood forests suggests that this may be a viable habitat for an EEEV vector. The primary vector of EEEV, *Cs*. *melanura*, is a hardwood swamp mosquito [3]. Although the upland hardwood forest is not classified as a wetland, it does contain mesic communities which are considered moderately moist sites [35]. Furthermore, the dense canopy cover reduces air circulation causing increased humidity within this land cover [39]. These conditions may provide adequate breeding sites for *Cs*. *melanura* thereby increasing the vector’s distribution among different habitats. Previous research has shown that in Florida *Cs*. *melanura* is evenly distributed across all habitat types, including hardwood forests [9].

Wetland hardwood forests, the habitat most often associated with the EEEV vector *Cs*. *melanura* in the northeast United States [3,40], ranked 12^th^ out of 14 in the compositional analysis of habitat use (Table 4). Furthermore, the median distance from cases was 696m compared to the state median of 654m. These results may indicate that wetland hardwood forests do not play as critical a role in the epizootic EEEV transmission cycle in Florida as it appears to play in the Northeastern states. This is supported by previous research of habitat associations with enzootic transmission which showed there was no association of EEEV transmission with wetland hardwood forests [9]. This may be the result of the vector *Cs*. *melanura* not being as confined to a specific habitat type in Florida [9]. Further studies need to be conducted to determine the affect wetland hardwood forests have on EEE transmission in Florida.

Finally, while this study implicates several habitats associated with EEE horse fatalities in Florida, there are other factors not analyzed that play a role in EEEV transmission and could explain, at least in part, the spatial patterns observed. Such a factor is the availability of a vaccine against EEEV for horses which requires semiannual boosters to ensure protection from EEE. However, vaccine usage is not tracked, and this adds a potentially confounding variable to the study if horse vaccination rates vary across the state. For example, if vaccination rates are lower in the Northern region, this could explain why there is such a high incidence of EEE horse fatalities despite low population densities. If future studies are able to explore vaccination rates, then researchers can better understand the role of habitat in EEEV transmission.

## Conclusions

Overall, the results of this spatial epidemiological study have demonstrated that EEE horse fatalities cluster in farmlands and rural residential lands that are located near wetlands and tree plantations. Identifying locations in Florida that exhibit these types of habitat configurations could ultimately be used to prevent EEEV transmission by targeting vector control measures in the highest risk areas. Future work might explore GIS-based models to predict EEEV transmission based on the results of this work. Furthermore, these findings are relevant to other locales with endemic EEEV that also have subtropical and tropical climates. For example, EEEV is endemic to both Central and South America and have endured epizootic outbreaks within their equine populations [41,42]. Despite human cases of EEE being quite low in South America, epizootic outbreaks have been known to affect thousands of horses [43]. By identifying high risk areas through habitat associations, targeted surveillance and prevention methods could be used to limit the impact EEEV has within the at risk populations of these countries, as well. Additionally, the approach used to identify spatial patterns and habitat associations of horse fatalities can be used to guide similar studies of other diseases.

In terms of Florida, specifically, this research highlights the potential importance of tree plantations in EEEV transmission. Tree plantations have been previously shown to be a habitat associated with an increased risk of enzootic EEEV transmission [9]. This study demonstrates that in Florida tree plantations are also a focus for epizootic transmission of EEEV. It appears both the abundance and proximity of tree plantations are factors associated with increased risk of EEE in horses and therefore humans. This association helps to explain why there is a spatially distinct difference in the amount of EEE horse cases across Florida. Tree plantations are scarce in southern Florida and despite having similar horse populations as the panhandle area, disease incidence is much lower. This study also associates upland hardwood forests with EEEV transmission. Again, both abundance and proximity play a role in increasing the risk of EEEV transmission to horses and humans. The focality of transmission was also highlighted in the local case cluster analysis. It is important to determine the ecological risk factors for EEEV transmission in Florida in order to reduce the number of human and horse cases. Furthermore, understanding the ecology of this disease will help to identify at risk areas, thereby providing better opportunities for vector control. By focusing on high risk habitats, prevention methods can be used to reduce the amount of disease transmission, resulting in better protection for both the equine and human populations in Florida and other areas where EEEV is endemic.

## Methods

### Study area

The state of Florida covers an area of about 170,304 km^2^. It is the only state with both subtropical and tropical regions. Florida is made up of five major land cover classes which collectively account for 94% of state’s habitat. These include wetlands (27%), upland forests (24%), agriculture (19%), urban (13%), and water (11%). Due to Florida’s high water tables, wetland areas tend to be fragmented and intermixed between other land cover classes, creating a complex mixed ecosystem [44].

GIS layers documenting Florida habitats were obtained from the state's five Water Management Districts. The Florida Department of Environmental Protection's Bureau of Watershed Restoration developed these land use and land cover maps using the Digital Ortho Quarter Quad Aerial Imagery program Color Infrared and True Color photography [45]. The schema of habitat classification descriptions for the land use-land cover encompassed four different levels, with Level 1 being the most basic and Level 4 the most specific [35]. In this study, ecological habitats were characterized using Level 2 land cover usage classifications. Level 2 descriptions were selected because they differentiated between various wetland types, as well as different residential features. The 42 sub-classifications found in the Level 2 categories were aggregated to 14 classes for use in this study: (1) Low Residential, (2) Crop and Pastureland, (3) Upland Hardwood Forest, (4) Vegetated Non-forested Wetland, (5) Tree Plantations, (6) Wetland Mixed Forest, (7) Upland Coniferous Forest, (8) Wetland Coniferous Forest, (9) Medium and High Density Residential, (10) Urban, (11) Water, (12) Wetland Hardwood Forest, (13) Shrub and Brushland, (14) Mining. The selected land use classifications were chosen based on their overall dominance and suspected habitat importance to equine populations and mosquito vectors associated with EEEV. For instance, certain water classes were combined (lakes, reservoirs, etc.), as were high and medium density residential classes. Tree crops and tree nurseries, low in abundance, were combined in the tree plantations class. The remaining classes were placed into an urban category (e.g. large paved areas, buildings, and airports). Coastal habitats were excluded from the study, since EEEV is only transmitted by freshwater mosquitoes.

### Horse cases

Florida had a total of 442 reported horse cases of EEE from 2005–2010 [46]. Case locations were georeferenced using GPS coordinates provided by the Florida Department of Health. Four cases in this database were excluded due to incomplete or missing coordinates, leaving 438 cases that were included in the analysis. Between 2005–2010, 54 out of 67 counties reported the occurrence of at least one horse case of EEE. Of the 10 counties with no horse cases of EEE, 8 were coastal counties. To establish the incidence of EEE horse cases, total equine populations were acquired for each county [33]. Cumulative disease incidence was then calculated by dividing the number of horse cases per county from 2005–2010 by the 2007 horse census population totals. The Spearman’s rank correlation coefficient was used to test if there was a relationship between the number of cases and the total population of horses in each county.

### Spatial analysis of EEE case clusters

To characterize the spatial pattern of EEE horse cases in Florida, the Density-Based Spatial Clustering of Applications with Noise (DBSCAN) technique was employed. DBSCAN is one of the most widely applied spatial clustering methods, since it can detect clusters of complex shapes and can operate at different spatial scales [47]. The algorithm works by moving point to point based on the (x,y) coordinates of each case and calculates the density-reachablity and point connectivity between cases; these values are then used to either assign points to particular cluster or designate them as statistical noise [47]. DBSCAN requires the user to specify two input parameters: the minimum number of points used to define a cluster (minPoints) and the neighborhood distance for defining clusters (epsilon). Two spatial clustering analyses were conducted using different DBSCAN parameters to examine both the regional and local clustering of cases. The parameters used to verify regional case clusters were a minimum of eight points and an epsilon distance of 25,000 meters for connectivity. Local clustering parameters were a minimum of four points and 6,000 meters for connectivity. Cases contributing to each of the clusters were examined by year to determine temporal disease patterns.

### Habitat analysis of EEE cases

A compositional analysis of habitat use, which is widely used in ecology to identify habitat use by wildlife [48-51], was conducted to rank which habitat types were most associated with cases of EEE in terms of proportional abundance [52]. A total of 14 aggregated classes, including; (1) Low Residential, (2) Crop and Pastureland, (3) Upland Hardwood Forest, (4) Vegetated Non-forested Wetland, (5) Tree Plantations, (6) Wetland Mixed Forest, (7) Upland Coniferous Forest, (8) Wetland Coniferous Forest, (9) Medium and High Density Residential, (10) Urban, (11) Water, (12) Wetland Hardwood Forest, (13) Shrub and Brushland, (14) Mining were used in the analysis. Habitats immediately neighboring EEE horse cases were compared to habitats in the surrounding landscape. Spatial scales ranging from 1-2km are commonly utilized to determine the spatial epidemiology of arthropod diseases [26,29,53]. The 1.5km distance was chosen because many of the bridge-vector mosquito flight ranges fall within this buffer range [54-56] and it has been successfully used in previous studies to determine landscape associations of enzootic EEEV activity in Florida [9]. Habitat proportions for each case were calculated from a 1.5km buffer around each individual site. Available habitats were calculated by considering the total habitat composition in the surrounding county [57,58]. The results of the analysis were summarized using a ranking matrix, which identified which habitats are proportionally most associated with EEE as compared to habitats available in the surrounding landscape.

### Distance analysis

**Figure 3:**
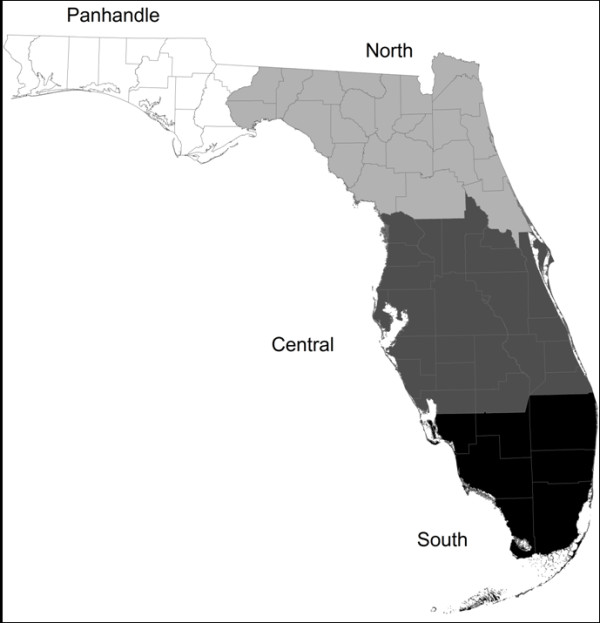
**Florida ecological regions.** The four ecological regions of Florida in this study include panhandle, north, central, and south.

A Euclidean distance analysis [59,60] was conducted to detect the proximity of each horse case of EEE to each of the 14 habitat classifications used in this study. Each individual horse case was used as a source point to calculate the distance (meters) to the nearest location in the landscape of each habitat type. These results were also used to compare the observed horse case distances to similar distances for all other locations in the surrounding landscape. Here, the surrounding landscapes were defined based on four ecoregions: Panhandle, North, Central, and South (Figure 3). The purpose of the division was to account for any regional ecological differences, so that each horse case is compared to other areas with similar habitat and landscape configurations. For each region, the median distance from horse cases to each habitat type was compared to the regional median using a Wilcoxon test. A nonparametric test was used because the distances for horse cases were not normally distributed. A Bonferroni correction was used in testing for statistical significance. The purpose of the comparison is to identify if horse cases are located closer to particular habitat types than would be expected for each region.

## Competing interests

The authors declare that they have no competing interests.

## Authors’ contributions

The author PVK was involved in the study conceptualization, research design, data analysis and the writing of the manuscript. JD contributed in the research design and data analysis and writing of the manuscript. LS supervised the data collection and contributed in reviewing the manuscript. RL and JA contributed in the data analysis. TRU served as the principal investigator, overseeing the overall direction of the project and contributed to the writing of the manuscript. All authors read and approved the final manuscript.
